# Activity in ventral premotor cortex is modulated by vision of own hand in action

**DOI:** 10.7717/peerj.88

**Published:** 2013-07-02

**Authors:** Luciano Fadiga, Luana Caselli, Laila Craighero, Benno Gesierich, Andriy Oliynyk, Banty Tia, Riccardo Viaro

**Affiliations:** 1 Department of Biomedical and Specialty Surgical Sciences, Section of Human Physiology, University of Ferrara, Ferrara, Italy; 2 Department of Robotics, Brain and Cognitive Sciences, Italian Institute of Technology, Genova, Italy

**Keywords:** Single-neuron recording, Grasping, Visuomotor, Frontal cortex

## Abstract

Parietal and premotor cortices of the macaque monkey contain distinct populations of neurons which, in addition to their motor discharge, are also activated by visual stimulation. Among these visuomotor neurons, a population of grasping neurons located in the anterior intraparietal area (AIP) shows discharge modulation when the own hand is visible during object grasping. Given the dense connections between AIP and inferior frontal regions, we aimed at investigating whether two hand-related frontal areas, ventral premotor area F5 and primary motor cortex (area F1), contain neurons with similar properties. Two macaques were involved in a grasping task executed in various light/dark conditions in which the to-be-grasped object was kept visible by a dim retro-illumination. Approximately 62% of F5 and 55% of F1 motor neurons showed light/dark modulations. To better isolate the effect of hand-related visual input, we introduced two further conditions characterized by kinematic features similar to the dark condition. The scene was briefly illuminated (i) during hand preshaping (pre-touch flash, PT-flash) and (ii) at hand-object contact (touch flash, T-flash). Approximately 48% of F5 and 44% of F1 motor neurons showed a flash-related modulation. Considering flash-modulated neurons in the two flash conditions, ∼40% from F5 and ∼52% from F1 showed stronger activity in PT- than T-flash (PT-flash-dominant), whereas ∼60% from F5 and ∼48% from F1 showed stronger activity in T- than PT-flash (T-flash-dominant). Furthermore, F5, but not F1, flash-dominant neurons were characterized by a higher peak and mean discharge in the preferred flash condition as compared to light and dark conditions. Still considering F5, the distribution of the time of peak discharge was similar in light and preferred flash conditions. This study shows that the frontal cortex contains neurons, previously classified as motor neurons, which are sensitive to the observation of meaningful phases of the own grasping action. We conclude by discussing the possible functional role of these populations.

## Introduction

Primates distinguish themselves from other species by a highly evolved grasping and manipulative capacity (MacNeilage, 1990; Sartori et al., 2012; Wells & Turnquist, 2001). Online visual guidance of hand and arm movements is one of the main prerequisites to their skilled hand use (Ma-Wyatt & McKee, 2007; Paillard, 1996). Grasping control mechanisms require synergistic activity of a visuomotor network comprising the anterior intraparietal area (AIP) of the lateral bank of the intraparietal sulcus and area F5 of the ventral premotor cortex (PMv; Jeannerod et al., 1995; Rizzolatti et al., 1996). Earlier studies support a major role of area AIP in encoding object 3D visual properties in a way suitable to guide grasping movements (Jeannerod et al., 1995; Murata et al., 2000; Sakata et al., 1995; Srivastava et al., 2009; Taira et al., 1990). In turn, area F5, which receives a relevant parietal input from area AIP (Rizzolatti & Luppino, 2001), would transform object representations into grasping motor commands further elaborated in the primary motor cortex (area F1; Fogassi et al., 2001). Consistent with their strong anatomical connections (Matelli, Luppino & Rizzolatti, 1985), parietal and ventral premotor areas share neuronal populations with similar visuomotor properties. Mirror neurons, discharging during the execution of a given motor act and during the observation of a similar motor act performed by others, were identified in area F5 (Gallese et al., 1996) and area PFG of the inferior parietal lobule (Fogassi et al., 2005; Fogassi et al., 1998). Besides, canonical neurons discharging during object grasping and object visual presentation were described in F5 (Fadiga et al., 2000; Murata et al., 1997; Raos et al., 2006), whereas neurons with similar properties were evidenced in AIP as “object-type visual-motor” neurons (Murata et al., 2000; Sakata et al., 1995; Taira et al., 1990). It has been suggested that these classes of neurons take part in processes of action-observation matching and sensorimotor transformation for grasping (Fadiga et al., 2000; Rizzolatti & Fadiga, 1998).

In addition to object- and action-related visuomotor neurons, Murata and co-workers described in area AIP a class of grasping motor neurons displaying a stronger response in light than in dark (“nonobject-type visual-motor” neurons; Murata et al., 2000). Since these neurons did not discharge during object fixation, the difference between light and dark conditions was interpreted as likely due to vision of own hand in action (Murata et al., 2000). In contrast with area AIP, in premotor area F5 there is no major evidence for motor neurons modulated by visual feedback of own hand action. In this study, we investigated in area F5 of behaving monkeys the presence of neurons with similar properties to AIP hand-related neurons. We discarded neurons with typical visual properties (mirror or canonical), focusing our attention on neurons showing motor properties only (i.e., movement-related activity both in light and dark environments). Moreover, we examined whether visual effects of the own acting hand are restricted to the AIP/F5 circuit, or if they extend to area F1 (Vigneswaran et al., 2013). As revealed by several anatomical (Gharbawie et al., 2011; Luppino & Rizzolatti, 2000; Matelli, Luppino & Rizzolatti, 1985; Rathelot & Strick, 2006) and electrophysiological studies (Graziano, Taylor & Moore, 2002; Hendrix, Mason & Ebner, 2009; Saleh et al., 2010; Umiltà et al., 2007), F1 displays neurophysiologically defined grasping-related regions which receive direct projections from F5 and represent the main descending component of the corticospinal projections to control wrist, hand and finger muscles.

One major issue of visual feedback studies is that possible differences of neuron activity could be caused by changes in kinematics between light and dark conditions rather than visual input. For this reason, in addition to the traditional light vs dark approach, we added two main modifications: (i) we investigated the effect of instantaneous visual presentation of the acting hand at different grasping phases, thus ensuring that arm/hand kinematics were not modified with respect to the dark condition, and (ii) the to-be-grasped object was always kept visible by a dim retro-illumination in order to reduce to a minimum the kinematic differences between light and dark.

## Materials and Methods

### Ethics statement

Experimental protocols were approved by the Veterinarian Animal Care and Use Committee of the University of Ferrara, by the Italian Ministry of Health and complied with the European laws on the use of laboratory animals (n. 08/2009). All surgery was performed under aseptic procedures and general anesthesia, and all efforts were made to minimize suffering. Monkeys were pre-medicated with atropine sulfate (0.1 mg/kg i.m.; MONICO SpA, Italy) and Zoletil 100 (20 mg/kg i.m.; Virbac Laboratories, France), and anesthetized by isoflurane (Abbott SpA, IL, USA) for the whole duration of surgery. Antibiotics and analgesics were administered postoperatively and experiments were started at least two weeks after the surgery.

### Basic procedures

Single-unit activity was recorded from areas F5 and F1 in three hemispheres of two behaving monkeys (*Macaca fascicularis*). Monkeys MK1 and MK2 (one female and one male, weighing 5.7 and 4.9 kg, respectively) were trained to perform a grasping task while sitting on a primate chair. After training, a recording chamber (diameter 30 mm) and a head-restraining device were surgically implanted on the left and right hemispheres of MK1 and on the left hemisphere of MK2. The position of the chambers (assessed before implantation using computer tomography and magnetic resonance scans) allowed us to record from a cortical region spanning area F1, the whole PMv (areas F4 and F5) and the caudal part of the frontal eye field (FEF).

### Electrophysiological recording

Single-unit recordings were performed using varnish-insulated tungsten microelectrodes with impedance 0.15–1.5 MΩ (measured at 1 kHz). During each experimental session, the microelectrode was inserted perpendicular to the cortical surface and was slowly advanced through the cortex by means of a microdrive (Kopf Instruments, CA, USA; step resolution: 10 µm). The recorded signal was amplified × 10000 (BAK Electronics, Germantown, MD, USA), filtered by a dual variable filter (0.3–5 kHz bandwidth; VBF-8, KEMO Ltd., Beckenham, UK), digitized (PCI-6071E, National Instruments, USA) at a sampling rate of 10 kHz and stored on a PC for off-line analysis. Action potentials were discriminated on-line by a dual voltage-time window discriminator (BAK Electronics, Germantown, MD, USA) and fed to an audio monitor (Grass Instruments, USA) to give the experimenter an auditory feedback on the neuron discharge during testing. The recording microelectrodes were also used for intracortical microstimulation (ICMS; train duration: 50–100 ms; pulse duration: 0.2 ms; frequency: 330 Hz; current intensity: 3–40 µA). Current intensity was controlled on an oscilloscope by measuring the voltage drop across a 10 kΩ resistor in series with the stimulating electrode. The accessible cortex was functionally explored through single-unit recordings and ICMS to assess the location of areas F1, F4, F5 and FEF. Criteria and functional characteristics described by Umiltà et al. (2001) were used to distinguish motor and premotor areas. Briefly, area F1 is characterized by low threshold of excitability (3–10 µA), robust discharge during active movements and response to somatosensory stimuli, area F4 is characterized by higher excitability threshold (10–40 µA), axial/proximal movements, discharge during tactile stimuli of face/body and response to visual stimuli within peripersonal space, and area F5 is characterized by an excitability threshold similar to area F4 (10–40 µA) but ICMS-evoked hand/mouth movements, discharge during goal-directed actions and response during observation of actions or objects.

### Naturalistic testing

Naturalistic tests were used to select neurons which were then examined through the experimental paradigm. Single-neuron activity was studied with reference to the execution of different hand/arm movements associated with different grip types or to the application of different sensory stimuli (Rizzolatti et al., 1990). In particular, grasping neurons were distinguished from reaching-related ones by presenting small pieces of food (raisins, apple, peanuts) at different distances and in different right/left locations. Visual properties were tested by showing to the monkey a series of hand actions (Gallese et al., 1996) and different objects (Murata et al., 1997; Raos et al., 2006). Specifically, we tested canonical properties by presenting food and 3D objects (pleasant or unpleasant) of different sizes, shapes and orientations to the monkey, whereas mirror properties were tested by putting, grasping, holding and manipulating food and 3D objects in front of the monkey. In addition, we also tested visual properties in particular conditions, e.g., mimicking grasping in the absence of object or performing actions with tools. Testing was performed by an experimenter at different distances and in different right/left locations. This functional characterization, together with ICMS data, allowed us to select hand-related motor neurons selective for precision grasping.

**Figure 1:**
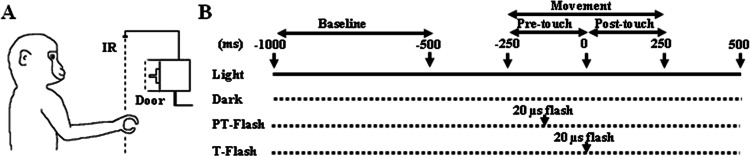
Experimental setup. (A) The monkey had to execute a precision grip in order to open the door of the food container. The target door, back-illuminated by a LED, was covered by an outer sliding door (Door) that the experimenter opened at each trial onset, giving the monkey a go-signal to start moving the hand from the resting position. The dashed line (IR) represents an infrared barrier positioned in front of the apparatus. (B) Time sequence of events during the grasping task and neuron recording. During the flash conditions, the scene was briefly illuminated by a single 20 µs xenon light flash delivered when the hand crossed an infrared barrier at 10 cm in front of the apparatus (PT-flash) or when both the thumb and index finger touched two small metal contact sensors attached to the sides of the to-be-grasped handle (T-flash).

### Grasping task

The pre-selected grasping neurons were studied by using a computer-controlled apparatus specifically designed to make the animal perform a reach-to-grasp task which naturally implied the execution of a precision grip in order to open the door of a small box and get a piece of food from the inside. The box was positioned at 30 cm in front of the monkey’s chest, at the height of the animal’s chin so that during the task, the monkey easily saw its own grasping hand (Fig. 1A). The precision grip was performed on a small plastic cube (side 8 mm) serving as a door handle and buried in a vertical groove to force a precision grip. To ensure that the movement was accurately executed under all conditions, the to-be-grasped cube was translucent and dimly back-illuminated by a red light-emitting diode (LED). LED intensity was kept low enough to prevent vision of the approaching fingers. Each trial began with the hand of the monkey positioned on a plane in front of its chest, at rest position. An external sliding door covering the to-be-grasped handle was remotely opened by the experimenter, giving the monkey a go-signal to initiate the grasping task. The task was performed under four different conditions (Fig. 1B), namely (i) grasping in light, when grasping was executed with continuous vision of the own movement, (ii) grasping in dark, when grasping was executed in absence of any visual information on the own movement, (iii) PT-flash condition, when grasping was executed in dark but with instantaneous visual feedback during the hand preshaping phase, and (iv) T-flash condition, similar to the PT-flash condition but with instantaneous visual feedback provided at hand-object contact. To exclude the systematic repetition of stereotyped movements, the order of presentation of the four conditions was randomized in each sequence and the time interval between two consecutive tasks was varied (usually in the 5–10 s range). Specifically, each sequence started with the light condition, that was followed by the dark, PT- and T-flash conditions (in random order), and ended with a second light condition (data not shown) to confirm the stability of neuronal activity and to verify that the three consecutive dark conditions (i.e., dark, PT- and T-flash) did not modify kinematics. During flash conditions, the scene was briefly illuminated by a single 20 µs-xenon light flash triggered by: (PT-flash) the signal of the hand crossing an infrared barrier at 10 cm in front of the apparatus or (T-flash) a signal delivered when both the thumb and index finger touched two small metal contact sensors attached to the sides of the to-be-grasped handle. The sensors used, i.e., E3FZ (Omron Europe BV, Hoofddorp, The Netherlands) for PT-flash and HEF4011B (NXP semiconductors, Amsterdam, The Netherlands) for T-flash, featured a latency time between signal and flash delivery of 1 ms for the infrared barrier cross (PT-flash) and 60–120 ns for handle touch (T-flash). The same trigger signals were also acquired to align neuronal discharges during the subsequent analysis.

### Kinematics recording

In separate sessions, grasping movements of MK1 were recorded with a 3D-motion optical analyzer (Qualisys Motion Capture System, Qualisys, Sweden). Three adhesive infrared-reflective spheres (diameter: 0.3 cm, weight: 0.04 g) were placed as markers on the upper limb skin over three anatomical landmarks: the wrist (head of the ulna) and the last-interphalangeal joint of the thumb and index finger. Three infrared cameras, placed at about 1.5 m from the monkey, were used to record the position of the markers. The motion analysis system provided 3D coordinates of the markers in space and time, enabling off-line reconstruction of the movement of each marker. Movements were recorded at a sampling rate of 240 Hz, a value considered more than appropriate for biological motion of primates (Saleh, Takahashi & Hatsopoulos, 2012). Wrist velocity and grip aperture (distance between the two markers placed on the fingers) were recorded in order to analyze the time of peak wrist velocity (measured from touch instant) and maximal grip aperture. Analyses were performed using Qualisys Track Manager software and custom MATLAB programs (MathWorks, Natick, MA, USA).

### Data presentation and statistical analysis

**Figure 2:**
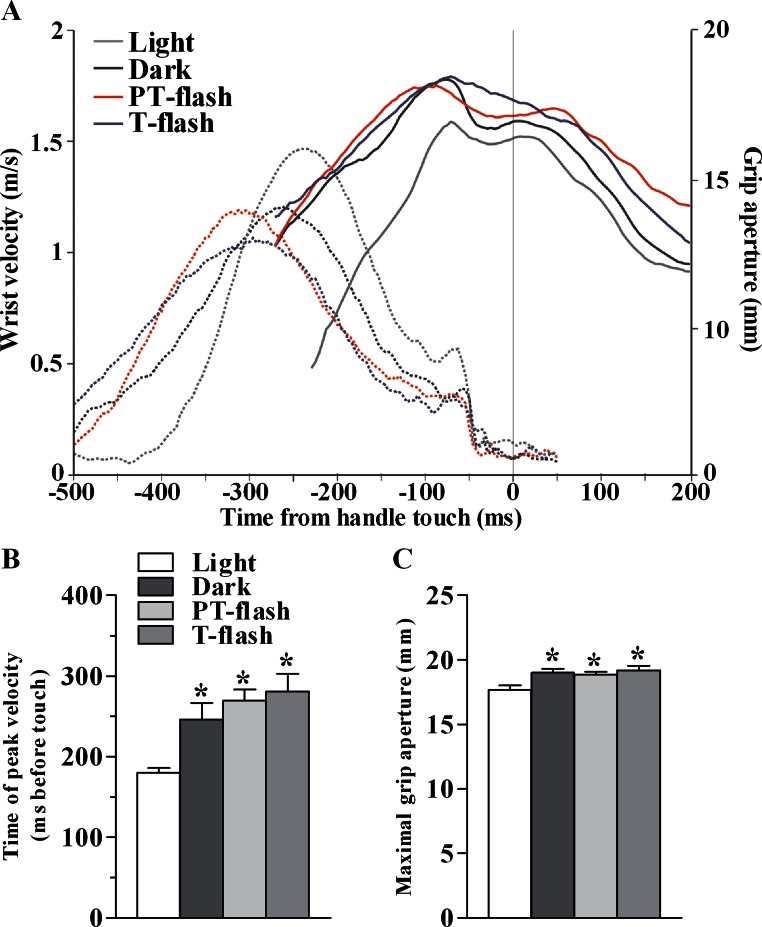
Kinematic features of grasping action in MK1. (A) Representative example of the time course (in ms) of wrist velocity (in m/s) and size of grip aperture (in mm) during the precision grip task in each condition during a single trial. (B) Time of peak wrist velocity (in ms) measured from handle touch (mean of all sessions). (C) Maximal grip aperture (in mm; mean of all sessions). *p < 0.05 different from light condition (Kruskal-Wallis test).

Differences in time of peak wrist velocity and maximal grip aperture among conditions (Fig. 2) were analyzed by the Kruskal-Wallis non-parametric test performed on the mean values across all sessions (*p* < 0.05).

Neural responses were analyzed as follows. To firstly ensure that the response of a selected neuron was modulated by hand grasping, the difference in activity between pre-movement (baseline, 500 ms period before movement start) and movement-related epoch (MOV, from 250 ms before handle touch to 250 ms after) was statistically assessed for each neuron by a two-way repeated-measures (RM) ANOVA with Epoch (two levels) and Condition (four levels) as factors. Only neurons showing significant differences were further taken into consideration. The single-neuron spike train, aligned with respect to handle touch and averaged, was convolved with a Gaussian-Kernel function (window width: 20 ms) to obtain a spike density function (SDF) which provided a continuous (1 ms-bin) time-dependent measure of firing patterns. Differences among conditions in individual neuron firing rate (Figs. 4 and 5) were assessed by a running two-tailed Student’s t-test performed on a 100 ms-bin stepped through the trial by 20 ms increments (*p* < 0.05). Moreover, to specifically study neuron sensitivity before and after finger contact with the object, epoch MOV was subdivided into two sub-epochs, namely pre-touch sub-epoch (from 250 ms before to object touch) and post-touch sub-epoch (from touch to 250 ms after it). To statistically validate the modulation of neuron discharge by the phasic visual presentation of the grasping hand, one-way RM ANOVAs with Condition (four levels) as factor, followed by Tukey’s LSD post-hoc comparisons (*p* < 0.05), were performed on normalized activity (to reduce inter-neuron variance) of flash-modulated neurons, considering peak discharge and mean discharge during epoch MOV (Fig. 6). Normalization was achieved for each neuron by dividing the SDF by the peak of maximal discharge across all conditions. Finally, to better characterize the properties of the considered flash-modulated neurons, an Ansari-Bradley dispersion test (*p* < 0.05) was applied to the temporal distribution of discharge peaks of normalized activity (Fig. 7).

## Results

### Kinematic evaluation of motor behavior

In order to verify that the transient visual feedback (flashes) provided in our experiment, did not constitute a significant perturbation, we assessed kinematic features of the grasping movement in all conditions, namely light, dark, PT-flash and T-flash. Figure 2A shows a representative example of time course of wrist velocity and grip aperture recorded in each condition for MK1 during a single trial. In all conditions, wrist velocity was characterized by a bell-shaped velocity profile and grip aperture predominantly increased during the pre-touch sub epoch (i.e., from 250 ms before touch to touch; see also Christel & Billard, 2002). Moreover, maximal grip aperture occurred after maximal wrist velocity and before hand-object contact. These results demonstrate that finger grasping movements (i.e., preshaping) began during the considered pre-touch sub-epoch. Considering all performed sessions, statistical analysis (Kruskal-Wallis test) performed on time of peak wrist velocity (Fig. 2B) revealed a significant right shift when the movement was performed in full light as compared to dark, PT-flash and T-flash conditions (*p* < 0.001). Similarly, Kruskal-Wallis test performed on grip size values (Fig. 2C) revealed that maximal grip aperture decreased (*p* = 0.019) in light condition compared to dark, PT- and T-flash conditions. Importantly, for both velocity and grip aperture, no statistical difference was present among dark, PT-flash and T-flash conditions.

**Figure 3:**
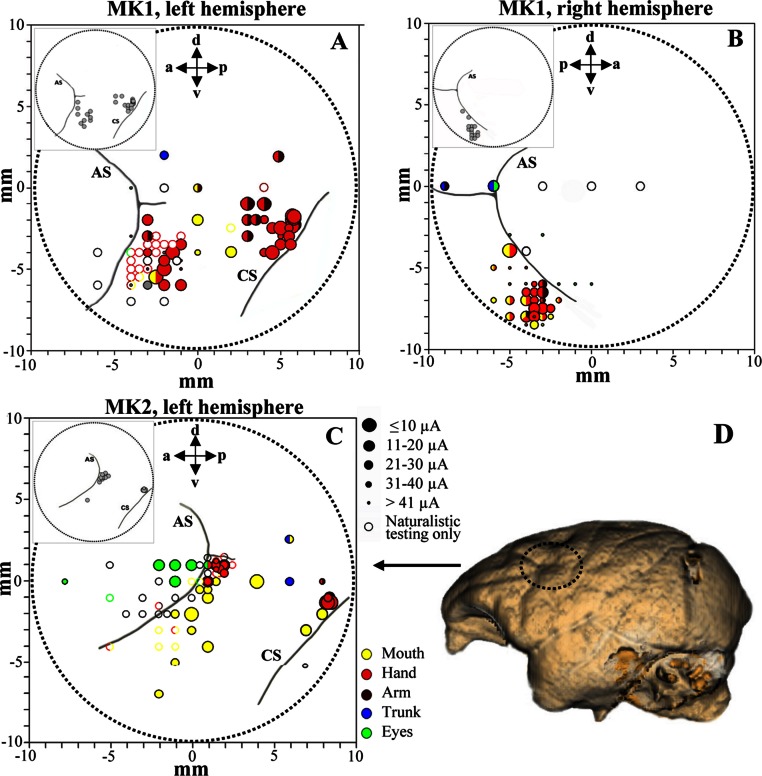
Penetration sites during experiments (A–B, MK1 and C, MK2) and example of brain surface reconstruction based on MRI data (D, MK2). The top left box represents the selected sites for single-unit recording. Filled symbols indicate movements evoked by ICMS at threshold current level. The size of the circles is correlated with threshold values. Colors refer to the specific movement evoked by ICMS. Unfilled symbols indicate sites not stimulated. AS, arcuate sulcus; CS, central sulcus.

### Single units and ICMS database

We analyzed 169 F5 neurons (102 from both hemispheres of MK1 and 67 from left hemisphere of MK2) and 128 F1 neurons (106 and 22 from the left hemispheres of MK1 and MK2, respectively). Functional maps are illustrated in Figs. 3A–3C. Figure 3D displays the reconstruction of the brain surface of MK2 (based on magnetic resonance imaging data) that was used to position the recording chamber on the skull (see Materials and Methods). Penetrations are marked according to the specific body-part movements associated with the recorded neuronal responses and the threshold current at which those movements were evoked by ICMS. All sites in the rostral bank of the central sulcus (area F1) were excitable with low threshold currents (MK1, 9.8 ± 0.8 µA; MK2, 11.4 ± 2.2 µA) evoking hand or finger movements. ICMS performed rostrally to F1 hand representation (estimated to be located in area F4) evoked face and axial movements at higher thresholds (MK1, 21.1 ± 5.9 µA; MK2, 27.9 ± 3.2 µA). Neurons in this region appeared to show large tactile receptive fields on the face and body, as well as visual peripersonal receptive fields around the tactile ones. The hand representation of area F5 was identified further rostrally, on the basis of distal movements evoked at threshold (MK1, 24.2 ± 2.8 µA; MK2, 28.2 ± 2.3 µA). Neuron discharge in this region was often related to goal-directed grasping actions. The presence of ICMS-induced eye movements at threshold (MK1, 25.9 ± 4.6 µA; MK2, 24.2 ± 5.7 µA) and the recording of saccade-related activity in a region anterior to area F5 and to the arcuate sulcus (AS) were considered as functional markers of the frontal eye field (FEF). Overall, the grasping-related activity of F5 and F1 neuronal samples recorded from the three hemispheres during the behavioral task was congruent with the functional characterization obtained through ICMS and naturalistic testing. In particular, we considered the F5 hand representation from the convexity of the cortex and the adjacent posterior bank of the inferior AS, namely F5c and F5p sectors respectively (Belmalih et al., 2009). All recording sites were identified in the first 4 mm from first detected activity. It is important to stress that all neurons considered henceforth were classified as purely motor grasping neurons, devoid of any visual response (i.e., significant increase of basal discharge) during mirror (Gallese et al., 1996) and canonical (Raos et al., 2006) property testing and devoid of any apparent non-grasping motor property (e.g., discharge associated with proximal movement and/or ICMS-evoked proximal movement).

### Grasping motor neurons are differently modulated by light and dark

**Figure 4:**
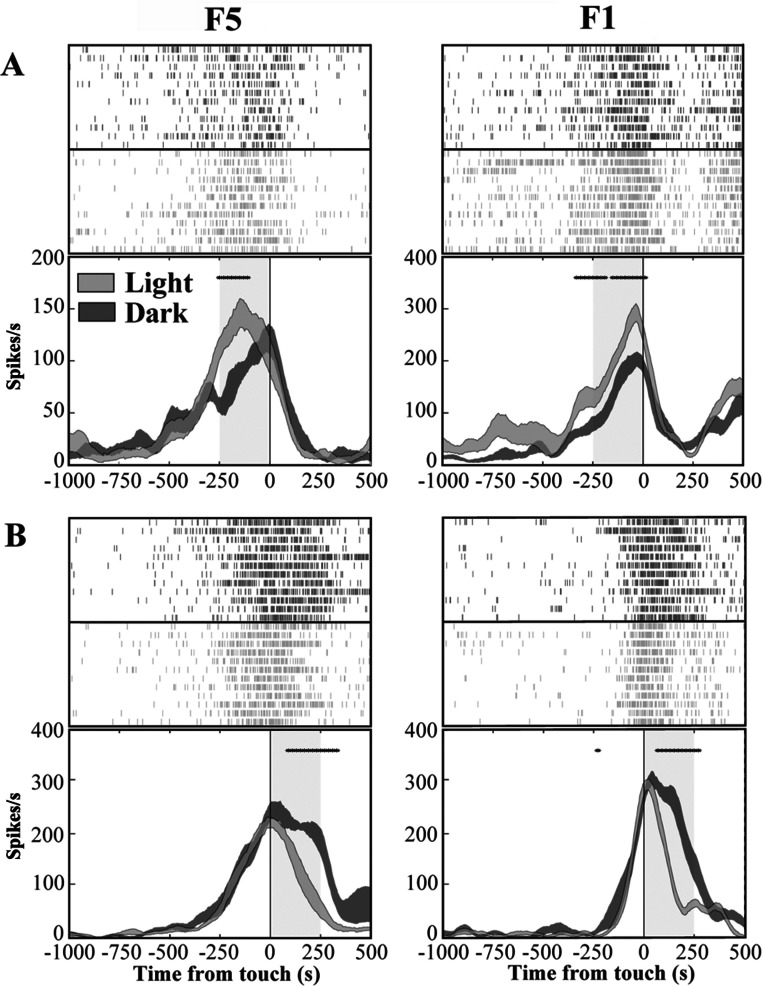
Activity of F5 and F1 motor neurons in light and dark conditions. (A) F5 and F1 neurons showing significantly higher SDF in light compared to dark condition, in the pre-touch sub-epoch. (B) F5 and F1 neurons showing significantly higher SDF in dark compared to light condition, in the post-touch sub-epoch. Rasters and SDF of 12 trials are aligned with respect to object touch. The thickness of SDF lines indicates the variability band (SEM). The vertical full line represents touch. *p < 0.05 different from light condition (running two-tailed paired Student’s t-test).

We investigated the presence, in areas F5 and F1, of neurons similar to the “nonobject-type visual-motor” neurons previously described in area AIP (Murata et al., 2000). In these neurons, grasping-related activity is significantly strengthened when the monkey observes its own hand action (light condition) with respect to a condition without visual feedback (dark condition). We therefore classified each grasping motor neuron of area F5 and area F1 according to the presence of significant differences between light and dark conditions as revealed by the running t-test performed on neuron discharges. The percentage of grasping motor neurons that responded differently in light and dark conditions (modulated neurons) was 62% (104/169) in area F5 and 55% (70/128) in area F1. The remaining non-modulated neurons were discarded from the study. Among modulated neurons, 36% (37/104) from F5 and 40% (28/70) from F1 showed higher activity in light than in dark (light-dominant neurons), whereas 64% (67/104) from F5 and 60% (42/70) from F1 displayed higher activity in dark condition (dark-dominant neurons). Figure 4 shows typical light- and dark-dominant neurons recorded from areas F5 and F1. The running t-test further revealed that, in both areas, the majority of light-dominant neurons were more active during the pre-touch sub-epoch (from 250 ms before object touch; 65%, 24/37, for area F5; 57%, 16/28, for area F1; Fig. 4A) whereas the majority of dark-dominant neurons were more active during the post-touch sub-epoch (from object touch to 250 ms after, 55%, 37/67 of F5 and 60%, 25/42 of F1 neurons; Fig. 4B).

### Grasping motor neurons are differently modulated by PT- and T-flash

**Figure 5:**
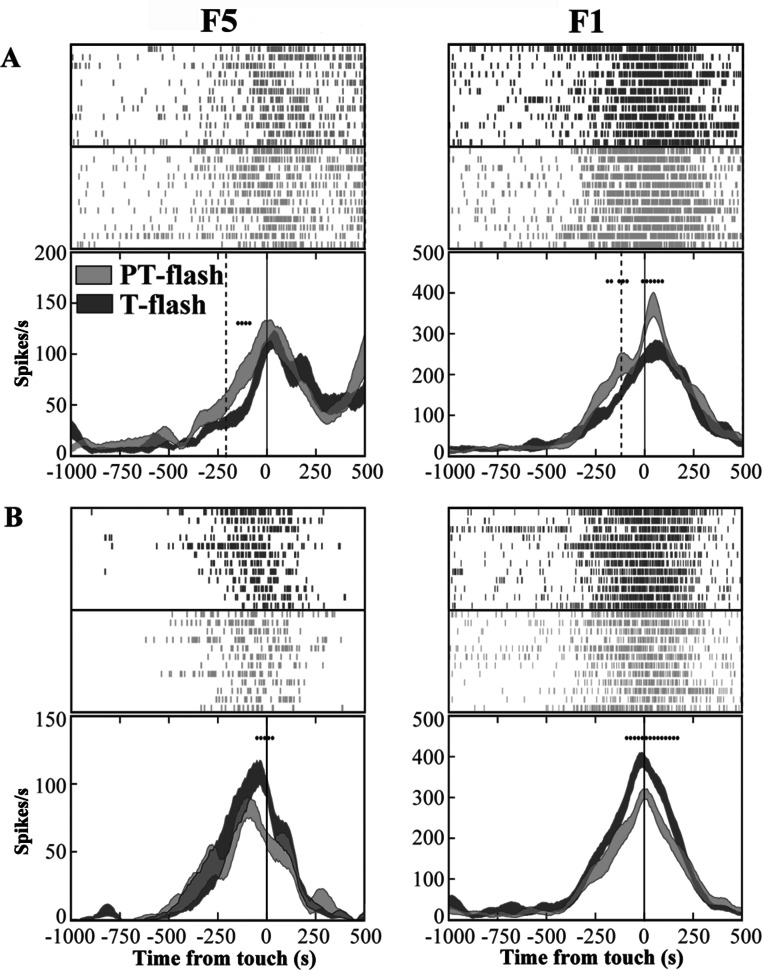
Activity of F5 and F1 motor neurons in PT- and T-flash conditions. (A) F5 and F1 neurons showing significantly higher SDF in PT-flash condition compared to T-flash condition. (B) F5 and F1 neurons showing significantly higher SDF in T-flash condition compared to PT-flash condition. Rasters and SDF of 12 trials are aligned with respect to object touch. Vertical lines represent touch (full line) or PT-flash delivery (dashed line). *p < 0.05 different from PT-flash condition (running two-tailed paired Student’s t-test).

In order to exclude an influence of the kinematics on neuron responses, two flash conditions (20 µs flash in dark condition), conveying a transient visual feedback, were introduced. We classified each grasping motor neuron of area F5 and area F1 according to the presence of significant differences between PT- and T-flash conditions, as revealed by the running t-test performed on the whole discharge. The percentage of neurons that were influenced by PT- or T-flash (flash-modulated neurons) was 48% (81/169) in area F5 and 44% (56/128) in area F1. The remaining non-flash-modulated neurons were discarded from the study. Among flash-modulated neurons, 40% (32/81) from F5 and 52% (29/56) from F1 showed higher activity in PT-flash than in T-flash condition (PT-flash-dominant neurons; Fig. 5A), whereas 60% (49/81) from F5 and 48% (27/56) from F1 displayed higher activity in T-flash condition (T-flash-dominant neurons; Fig. 5B).

### Flash populations present a specific activity profile

**Figure 6:**
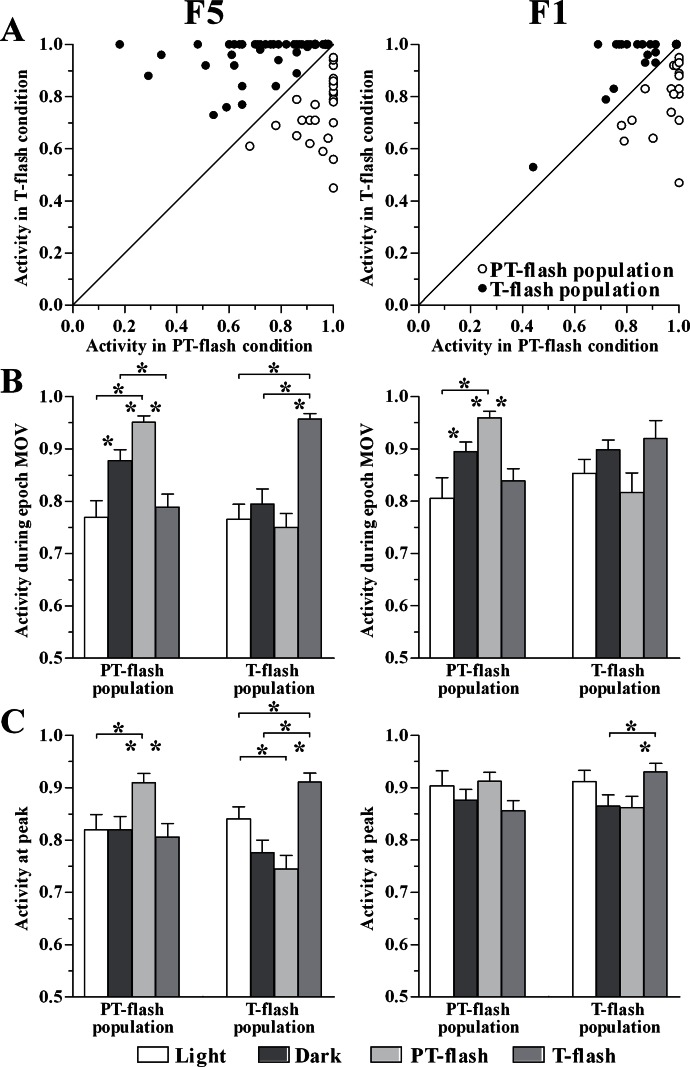
Activity of PT- and T-flash populations in all conditions. (A) F5 and F1 normalized individual neurons activity during epoch MOV in PT- and T-flash conditions. (B) F5 and F1 normalized population activity during epoch MOV showing modulation in a condition-dependent manner. (C) F5 and F1 normalized population activity at peak showing modulation in a condition-dependent manner. *p < 0.05 different from other conditions (ANOVA followed by Tukey’s LSD post-hoc test).

The normalized activity of individual PT- and T-flash dominant neurons (Table 1) in PT- and T-flash conditions was reported (Fig. 6A). In order to characterize the properties of these neuronal classes and to verify that they constitute distinct populations, we studied their activity by comparing all conditions (i.e., light, dark, PT- and T-flash). We firstly analyzed the normalized activity during epoch MOV (Fig. 6B). Considering F5, the ANOVA performed on PT- and T-flash-dominant neurons revealed that these neurons increased firing in the preferred flash condition with respect to all other conditions (*F*_3,93_ = 12.11, *p* < 0.001 and *F*_3,144_ = 16.90, *p* < 0.001, respectively). Considering F1, the ANOVA performed on PT-flash-dominant neurons revealed that firing strongly increased in PT-flash condition as compared to other conditions (*F*_3,84_ = 7.76, *p* < 0.001) whereas the ANOVA on T-flash-dominant neurons revealed no difference among conditions (*F*_3,78_ = 2.69, *p* = 0.052). In brief, the analysis of mean discharge during movement revealed that both flash populations are clearly represented in F5, while only the PT-flash population is distinguished in F1.

**Table 1:** Number of light-, dark-, PT- and T-flash-dominant neurons.

	PT-flash- dominant	T-flash- dominant	Non-flash- modulated	Total
F5				
Light-dominant	4	10	23	37
Dark-dominant	14	21	32	67
Non-modulated	14	18	33	65
Total	32	49	88	169
F1				
Light-dominant	3	7	18	28
Dark-dominant	11	3	28	42
Non-modulated	15	17	26	58
Total	29	27	72	128

In parallel, we studied the normalized peak discharge (Fig. 6C). Considering F5, the ANOVA performed on PT- and T-flash-dominant neurons revealed that these neurons had higher peak value in the preferred flash condition compared to all other conditions (*F*_3,90_ = 3.65, *p* = 0.016 and *F*_3,144_ = 10.54, *p* < 0.001, respectively). By contrast, in F1 the ANOVA performed on PT-flash-dominant neurons revealed no difference among conditions. T-flash-dominant neurons showed only a higher peak value in T-flash than in PT-flash and dark conditions (*F*_3,75_ = 3.00, *p* = 0.036). To summarize, similarly to the analysis performed on epoch MOV, both flash populations are clearly represented in F5, while they are not distinguished in F1.

**Figure 7:**
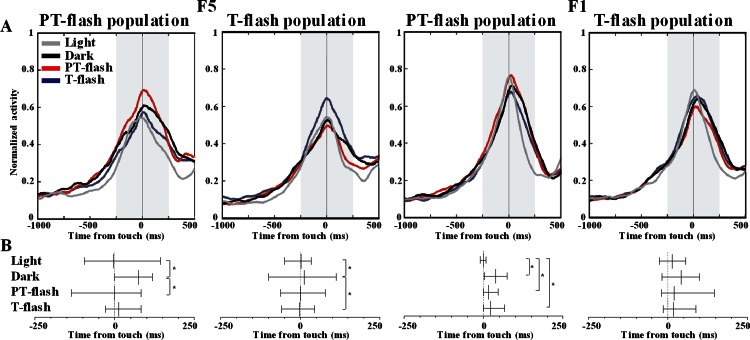
Peak time distribution of PT- and T-flash populations in all conditions. (A) Average normalized activity of F5 and F1 flash populations. (B) Temporal distribution of peak activity of F5 and F1 flash populations. Vertical dotted lines represent touch. The small vertical line crossing each sample is the median. Whiskers define the inter-quartile-range (IQR). *p < 0.05 different from other conditions (Ansari-Bradley dispersion test).

To further disclose the properties of F5 and F1 flash-populations, we analyzed the temporal distribution of discharge peaks in all conditions. The average normalized activity of F5 and F1 flash populations is represented (Fig. 7A). The Ansari-Bradley dispersion test revealed that peak distribution of F5 flash populations varied according to the flash condition for which these neurons expressed selectivity (Fig. 7B). Peak dispersion of flash populations was significantly different between light and dark conditions (*W*^∗^ = 2.0, *p* = 0.046 and *W*^∗^ = 2.9, *p* = 0.003, respectively for PT- and T-flash populations). Importantly, dispersion in the preferred flash condition was similar to light and different from dark condition (*W*^∗^ = 2.1, *p* = 0.038 and *W*^∗^ = 2.0, *p* = 0.049, respectively for PT- and T-flash populations). In F1, peaks dispersion of flash-dominant neurons presented a different pattern from F5. Considering PT-flash population, the Ansari-Bradley test revealed no major differences among dark, PT-flash and T-flash conditions, whereas dispersion in light condition significantly differed from dark (*W*^∗^ = 3.2, *p* = 0.001), PT- (*W*^∗^ = 3.0, *p* = 0.003) and T-flash (*W*^∗^ = 2.7, *p* = 0.008) conditions. Considering T-flash-dominant neurons, no difference was found among conditions. In short, this temporal analysis revealed that F5 flash populations presented a similar peak time distribution in light and preferred flash conditions. This pattern was not confirmed in F1, which displayed similar peak dispersion in flash and dark conditions.

## Discussion

The present study investigated, in frontal areas F5 and F1, the presence of neurons sensitive to the observation of the own grasping action. Our results revealed that both areas contain populations of neurons which activity is modulated by continuous (light condition) as well as transient (flash conditions) visual feedback. Since grasping kinematics did not differ between dark and flash conditions, we confirm that differences in neuron activity were due to hand-related visual input rather than to different motor strategy. In the following sections, we discuss the functional characterization and possible role of these populations in the two areas.

### Continuous vision of own acting hand affects grasping motor neurons

Among F5 and F1 motor neurons, two different classes of neurons, namely light- and dark-dominant neurons, were found to discharge differently depending on the presence/absence of visual feedback, i.e., vision of the hand or hand-object interaction (Nelissen et al., 2011; Raos et al., 2006). Note that, unlike a similar study conducted in area AIP (Murata et al., 2000), in our paradigm the target object remained visible in all conditions and, therefore, vision of object did not constitute a discriminating factor. Light-dominant neurons were mostly represented in the pre-touch phase (hand preshaping) although visual information was available throughout the entire action. This evidence is in agreement with that reported by Murata et al. (2000) who suggested a major role for these neurons in encoding the pattern of hand movements during handgrip formation. However, light dominance could also be attributed to the modulation of action kinematics by online visual feedback (Churchill et al., 2000; Rand et al., 2007; Schettino, Adamovich & Poizner, 2003; Winges, Weber & Santello, 2003). On the other hand, dark-dominant neurons were more represented in the post-touch phase. Post-touch dark-dominance was probably related to finger posture corrections ensuing proprioceptive, tactile and force feedbacks following hand-object contact (Nelissen et al., 2011; Raos et al., 2006). Discharge after touch could also be due to signaling of hand position in the peripersonal space (Buneo & Andersen, 2012).

Overall, this analysis demonstrates that F5 and F1 contain neurons, apparently indistinguishable from motor neurons, which are modulated by vision of the own acting hand.

### Transient vision of own acting hand affects grasping motor neurons

Since light dominant property mainly occurred in the pre-touch sub-epoch, underlining the importance of vision of the hand in this phase, we introduced two additional conditions providing transient visual feedback during hand preshaping or at hand-object contact (PT- or T-flash, respectively). Among F5 and F1 grasping motor neurons, two different classes, namely PT- and T-flash-dominant neurons, discharged differently depending on the instant at which visual feedback was provided. It is worth noting that T-flash condition is related to an effective contact with the object, whereas PT-flash condition is associated with distant spatial locations for the hand and object (see also Fluet, Baumann & Scherberger, 2010). Hence, our results support the idea that F5 and F1 regions play an important role for integration of context information for grasping (Fluet, Baumann & Scherberger, 2010). Moreover, we can speculate, in line with computational studies (Bays & Wolpert, 2007; Miall, 2003; Wolpert & Ghahramani, 2000), that visual feedback of a meaningful phase of the own ongoing action specifically reinforces online the motor program involved in that particular action. To be more precise, flash-responsive neurons may contribute to distinguish between own programmed actions and external events by matching predicted and actual visual feedback from own actions (Bays & Wolpert, 2007).

Overall, this analysis demonstrates that F5 and F1 cortices contain neurons, apparently indistinguishable from motor neurons, which are modulated by vision of own hand in a particular phase of action, namely preshaping and touch.

### Two populations sensitive to vision of own hand are clearly represented in F5

By comparing the four conditions, we confirmed that F5 flash-dominant neurons constitute sub-populations of motor neurons characterized by higher activity (during movement and at peak) in the preferred flash condition as compared to other conditions. In particular, since grasping performance in dark and flash conditions was characterized by similar kinematic features, changes in firing rate among these conditions cannot be attributed to different motor strategies and are likely due to the vision of own hand in action. In addition, these populations displayed, in the preferred flash condition, a peak dispersion similar to light and different from dark condition. By contrast, in F1, flash populations were not clearly represented, albeit PT-flash population property emerged when considering overall movement discharge.

In agreement with our results, a previous fMRI study in humans (Ehrsson, Spence & Passingham, 2004) evidenced a key role of the premotor cortex in bodily self-attribution, as revealed by increased activity during vision of own (or considered as own) hand. It can be argued that visual feedback of own hand action modulates F5 directly, since it is known that this area contains visuomotor neurons responding to observation of goal-direction actions, albeit this property has only been detected when observing other individuals (Rochat et al., 2010). Alternatively, given the rich connections between AIP and F5 (Matelli, Luppino & Rizzolatti, 1985), the activity of F5 flash populations could reflect direct AIP modulation by visual feedback.

Considering F1, previous reports in monkeys confirm that this area presents some observation-evoked responses (Dushanova & Donoghue, 2010; Tkach, Reimer & Hatsopoulos, 2007; Vigneswaran et al., 2013). Consistently, significant changes in activity of human area 4 during action observation have been reported in several imaging and electrophysiological studies (Caetano, Jousmaki & Hari, 2007; Fadiga et al., 1995; Muthukumaraswamy & Johnson, 2004). The modulations of F1 activity have been proposed to reflect the strong cortico-cortical interconnections with area F5 (Cerri et al., 2003; Dum & Strick, 2005; Fadiga et al., 1995; Kilner & Frith, 2007; Schmidlin et al., 2008; Shimazu et al., 2004). On the other hand, since we found that flash and dark conditions presented similar peak dispersions in F1, modulation in this area could be related to grasping kinematic features (Hendrix, Mason & Ebner, 2009; Vargas-Irwin et al., 2010).

As evidenced by our results, visual information conveyed by the brief illumination of own hand action affected more consistently F5 than F1 neuron response. A possible explanation for this difference between the two areas could be a distinct contribution to the analysis of motor-relevant visual feedback. Accordingly, previous experiments (Umiltà et al., 2007) described different time-courses of discharge in F5 and F1 during grasping, F5 being involved in an early phase and F1 in all phases of action. In fact, along F5/F1 intracortical pathways, visual information on the performed movement is transformed from an extrinsic reference framework (F5) which defines the spatial position of the hand (with respect to the object) to an intrinsic framework (F1) based on muscle and joint space to generate accurate grasping (Kakei, Hoffman & Strick, 2003). Alternatively, the difference described between F5 and F1 could be attributed to a modulation/gradient effect along the parietal-frontal pathway (Gharbawie et al., 2011). Although we found a clear distinction between the two areas, a minor similar effect was found in F5 and F1 PT-flash populations, i.e., a stronger activity in dark compared to light condition. This modulation is not surprising, considering that it could reflect finger posture adjustments derived from proprioceptive feedback for accurate hand preshaping. This activity could be further reinforced when transient visual feedback relative to the pre-touch phase is provided.

### Possible relation with other visuomotor properties

This study demonstrates for the first time that the monkey frontal cortex contains, mainly in F5, neurons previously classified as motor neurons, which are sensitive to the observation of meaningful phases of the own grasping action. Observation of the agent’s own acting effector has been proposed as a fundamental step in the biological process leading to the neuronal activation associated to the observation of actions performed by others (Heyes, 2010; Keysers & Perrett, 2004; Murata & Ishida, 2007; Rizzolatti & Fadiga, 1998). In particular, the parietofrontal mirror system (Cattaneo & Rizzolatti, 2009) may develop from the observation of one’s own hand action, seen from different perspectives. Through the visual feedback system normally guiding action execution, motor invariance would be extracted, creating a match between action and vision that could be generalized to the observation of actions executed by other individuals. Further experiments are required to explore this hypothesis, e.g., studying these neurons when visual information is disrupted and extending testing to mirror neurons.
